# Multimodal optical imaging combining voltage-sensitive dye ElectroFluor630 with genetically encoded calcium, glutamate, or voltage indicators

**DOI:** 10.1117/1.NPh.13.S2.S23207

**Published:** 2026-06-03

**Authors:** Katarina D. Milicevic, Amal M. Abdulkadir, Kaio S. Tom, Corey D. Acker, Ping Yan, Yukun A. Hao, Sungmoo Lee, Lan X. Liu, Michael Z. Lin, Leslie M. Loew, Srdjan D. Antic

**Affiliations:** a UConn Health, School of Medicine, Department of Neuroscience, Farmington, Connecticut, United States; b University of Connecticut, Institute for the Brain and Cognitive Sciences (IBACS), Storrs, Connecticut, United States; c University of Connecticut Health Center, Technology Incubator Program (TIP), Potentiometric Probes LLC, Farmington, Connecticut, United States; d Stanford University, Departments of Neurobiology, Bioengineering, and Chemical and Systems Biology, Stanford, California, United States; e University of Connecticut School of Medicine, Richard D. Berlin Center for Cell Analysis and Modeling, Farmington, Connecticut, United States

**Keywords:** ElectroFluor630, GCaMP6f, iGluSnFR, ASAP2s, ASAP5-Kv

## Abstract

**Significance:**

Combining genetically encoded neuronal activity indicators (GENIs), restricted to only one neuron type, with a voltage-sensitive dye (VSD) that reports pan-neuronal activity, could be beneficial for understanding neural circuits. Recently introduced far-red EF-630 may be compatible with green GENIs and could serve as an internal reference of neuronal activity for multiple GENIs.

**Aim:**

Here, we assess the EF-630 compatibility with several green-fluorescent-protein-based GENIs, including the calcium indicator GCaMP6f, the glutamate indicator iGluSnFR, and two voltage indicators ASAP2s, and ASAP5-Kv, for recording neuronal aggregate responses.

**Approach:**

Mouse brain slices expressing each GENI were stained with EF-630, and then extracellular stimulation and population optical imaging were sequentially performed at two wavelengths. In addition, cre-dependent ASAP5-Kv transgenic mice were generated and characterized.

**Results:**

Dual recordings provided population signals in both channels for all combinations. For each indicator pair, we quantified ΔF/F amplitudes and compared ON and OFF kinetics. ASAP2s and ASAP5-Kv displayed faster temporal dynamics and less temporal summation than VSD signals, suggesting the influence of cell-type-specific expression in observed kinetics.

**Conclusions:**

These kinetic differences underscore how both the choice of indicator pair and the targeted cell type influence the interpretation of neural population activity. Overall, our work provides the first systematic characterization of paired VSD-GENI measurements, establishing practical considerations and performance benchmarks for dual optical imaging of neuronal populations.

## 1 Introduction

Experimental measurements of neuronal activity are essential for understanding brain function in both health and disease. The depth and rigor of such analyses depend critically on the quality and breadth of the underlying experimental recordings. Here, we define quality primarily in terms of signal-to-noise ratio (SNR), and breadth as the number of informative physiological parameters that can be interrogated through experimental design. With respect to breadth, it has been well established that observing the same biological process (e.g., brain region activity) from multiple complementary perspectives,^1^^,^^2^ for example, using both calcium and voltage measurements, can be highly advantageous.^1^^,^^3^ When two indicators (e.g., calcium and voltage) are used in the same neuron, each reports a distinct aspect of the same underlying physiological process.^4^ When such indicators label different cell types (e.g., excitatory versus inhibitory neurons), they enable more precise interrogation of neural circuit interactions. For example, in the mouse olfactory bulb, axon terminals of olfactory sensory neurons were labeled with the red-emitting calcium indicator Cal-590, whereas the receptive dendrites of mitral cells within the same olfactory glomerulus were labeled with the green-emitting voltage indicator ArcLightD.^5^ Dual calcium and voltage recordings from individual olfactory glomeruli provided a unique opportunity to dissociate input and output processes, specifically: (a) olfactory receptor neuron nerve terminals (input) and (b) mitral/tufted cell apical dendrites (output).^5^ At present, examples of combined calcium–voltage imaging are numerous^6^^–^^8^ and span diverse research fields, from cardiac physiology^9^ to studies of dendritic and axonal physiology in central nervous system neurons.^4^^,^^10^

Genetically encoded neuronal activity indicators (GENIs) have become indispensable tools in systems neuroscience.^11^^–^^13^ Their principal advantages include: (a) cell-type-specific labeling; and (b) noninvasive expression, achieved either through a transgenic animal approach,^14^ or intravenous adeno-associated virus (AAV) delivery.^15^ Voltage-sensitive dyes (VSDs) enable labeling across large brain regions and therefore remain widely used for probing brain activity.^16^^,^^17^ Relative to GENIs, VSDs offer two key benefits: (a) faster optical response times^18^ and (b) substantially lower experimental cost.^19^

Recently, the VSD ElectroFluor630 (EF-630) was developed, featuring sub-0.1-ms kinetics and far-red emission (>665  nm),^6^^,^^20^ thereby enabling potential combination with indicators that utilize green fluorescent protein (GFP). When paired with cell-type restricted green indicators (GENIs), EF-630 could report unrestricted, pan-neuronal activity. Its rapid kinetics also make it well-suited to serve as an internal reference for comparative evaluation of different GENI classes.

In this study, we evaluated the compatibility of EF-630 with several GFP-based genetically encoded indicators, including calcium indicators (GECIs), glutamate indicators (GEGIs), and voltage indicators (GEVIs). Specifically, we tested four GFP-based proteins: GCaMP6f, iGluSnFR, ASAP2s, and ASAP5-Kv. Our objective was to determine whether functional signals from EF-630 and a GFP-based GECI, GEGI, or GEVI could be independently and reliably recorded from the same region of interest (ROI), and to assess both the feasibility and performance of such dual-mode imaging. Across all indicator pairings, dual-modality recordings consistently yielded detectable population-level signals in both channels. For each pairing (e.g., VSD + GEVI), we compared ΔF/F amplitudes, evaluated whether the optical signals exhibited matched or opposite polarities, and quantified differences in ON and OFF kinetics among reporters. Relative to EF-630, signals from GEVIs (ASAP2s and ASAP5 selectively expressed in striatal D1 spiny neurons) exhibited larger response amplitudes and slightly faster decay kinetics, leading to an apparent increase in temporal summation in the EF-630 channel compared with the GEVI channel. Given the well-characterized faster kinetics of EF-630, these results likely demonstrate the effects of cell type-restriction in the expression of GEVIs. Although EF-630 and GEVIs differ in kinetics, simultaneous measurements from the same ROI allow EF-630 to serve as a reference signal for correcting trial-to-trial variability unrelated to indicator performance. This improves comparisons between different GENIs expressed in the same neuronal population. Collectively, these results provide a systematic characterization of dual-indicator performance, demonstrating the complementary strengths of combining a voltage-sensitive dye with GFP-based protein indicators for neuronal population imaging.

## 2 Materials and Methods

Experiments were done according to the animal protocol (AP-200902) approved by the UConn Health Institutional Animal Care and Use Committee (IACUC).

### 2.1 Dye Synthesis and Dye Labeling

Voltage-sensitive dye, “EF-630” with a chemical name “Di-4-ANEQ(F)PTEA”, was synthesized in our laboratory following the steps described in Refs. 19, 21, 22. Some additional amounts of EF-630 were purchased from Potentiometric Probes, Farmington, Connecticut, United States.

Brain slices were stained with EF-630 directly in the recording chamber, which was equipped with a flat, 25-mm-diameter glass bottom. After transferring a slice into the chamber, it was perfused with artificial cerebrospinal fluid (ACSF) for several minutes. Perfusion was then temporarily halted to allow dye application. To localize the dye and minimize reagent use, a stainless-steel ring (inner diameter ∼10  mm) was placed around the slice, creating a confined ACSF pool (∼500  μL). Into this pool, 20  μL of a 166  μM EF-630 stock solution was added, resulting in a final dye concentration of ∼6.64  μM. Following a 45-s staining period, perfusion with dye-free ACSF (0.5 mL/min) was resumed for 4 min to wash out unbound dye. Optical recordings commenced ∼5  min after dye application, and slices consistently yielded stable signals for at least 60 min thereafter.

### 2.2 Transgenic Animals

All animals were housed in standard conditions with free access to food and water, in a 50% dark/light cycle.

**D1-Cre** mouse line (MMRC Stock: 030989-UCD) was obtained from Jackson Labs.

#### 2.2.1 ASAP2s mouse

Generation of the Ai169-ASAP2s mouse line (Jax Lab #:031569) harboring TIT2L-ASAP2-ICL-tTA2, a Cre-dependent ASAP2s gene at the TIGRE2.0 locus, was previously described.^23^ Initially, transgenic ES cells of a 129S6/SvEvTac x C57BL/6 F1 background were used to generate chimeric mice, which were bred to PhiC31-expressing C57BL/6 transgenic mice to establish germline founders while removing an AttB/AttP-flanked PGK-hygro-SV40polyA cassette in the transgene. These F1 founders were outcrossed to C56BL/6 three times, and F4 mice with TIT2L-ASAP2-ICL-tTA2 but lacking the PhiC31 gene were crossed to generate TIT2L-ASAP2-ICL-tTA2 homozygotes. These TIT2L-ASAP2-ICL-tTA2 homozygotes were then crossed with a D1-Cre mouse line (MMRC Stock: 030989-UCD) to generate mice expressing ASAP2s in D1 receptor-expressing spiny projection neurons of striatum (D1-SPN).

#### 2.2.2 ASAP5-Kv mouse

To generate the Cre-dependent ASAP5-Kv mouse line, a donor plasmid was constructed with a CAG-loxP-SV40polyA-loxP-ASAP5-Kv-SV40polyA (CAG-LSL-ASAP5-Kv) expression cassette flanked by attB recombination sites, where CAG is a cytomegalovirus enhancer with a chicken beta-actin promoter, Kv is the proximal retention and clustering segment of Kv2.1, and SV40polyA is the simian virus 40 polyadenylation signal. This donor plasmid was co-transfected with a PhiC31 integrase expression plasmid into blastocysts derived from C57BL/6 mice harboring an attP landing site within the H11P locus. Resulting chimeric mice were bred with C57BL/6 mice to establish germline-transmitting founder lines. Cre-dependent expression of ASAP5-Kv was achieved by crossing homozygous CAG-LSL-ASAP5-Kv transgenic mice with appropriate Cre driver lines, thereby restricting GEVI expression to defined neuronal populations.

### 2.3 AAV Injections

Two AAV vectors were purchased from Addgene: GCaMP6f-FLEX (pAAV-CAG-Flex-mRuby2-GSG-P2A-GCaMP6f-WPRE-pA (AAV1), catalog number #68719), and iGluSnFR (pAAV.hSynapsin.SF-iGluSnFR.A184S (AAV9), catalog number #106174).

AAV injections into the mouse striatum were performed under continuous isoflurane anesthesia using an R610 Veterinary anesthesia machine (RWD, Shenzhen, Guangdong, China). Virus was delivered with an R462 syringe pump (RWD) connected to a Hamilton syringe fitted with a 33-gauge metal needle, at a rate of 50 nL/min. The injection volume varied in the range of 100 to 200 nL. Typical stereotaxic coordinates relative to the bregma were used (striatum: AP +0.5  mm, ML ±1.5  mm, DV −3.0  mm; cortex: AP +2.68, and ML ±1.0, DV −1.5), with minor adjustments based on age and size. After injection, the needle was left in place for 5 min to minimize reflux before being slowly withdrawn.

### 2.4 Optical Imaging

Following deep anesthesia with isoflurane, mice of both sexes (P35-P60) were decapitated in accordance with institutionally approved animal care and use protocols. Brains were rapidly extracted into ice-cold, oxygenated saline containing (in mM): 125 NaCl, 26 ${\mathrm{NaHCO}}_{3}$, 2.3 KCl, 1.26 ${\mathrm{KH}}_{2}{\mathrm{PO}}_{4}$, 2 ${\mathrm{CaCl}}_{2}$, 1 ${\mathrm{MgSO}}_{4}$, and 10 glucose. The coronal brain slices (300  μm) were cut from the frontoparietal cortex, incubated at 34°C for 15 min, and subsequently maintained at room temperature until use. For imaging experiments, acute slices were transferred to an Olympus BX51WI upright microscope equipped with a 10× objective (0.3 NA) and continuously perfused with aerated saline (95% $O_{2}/5\%$ ${\mathrm{CO}}_{2}$). All experiments were conducted at a controlled temperature of 34±1°C. Synaptic stimulation was delivered via a stimulus isolation unit (IsoFlex, A.M.P.I., Jerusalem, Israel). Stimulation electrodes were fabricated from borosilicate glass capillaries with filament (1.5 mm outer diameter; resistance ∼2  MΩ) and backfilled with saline. Electrical stimuli (1 ms duration, 135 nA) were applied in trains of three pulses at interstimulus intervals (ISI) of 120 ms (8.3 Hz), 50 ms (20 Hz), or 12 ms (83 Hz). Optical trials were typically 3 s in duration, corresponding to continuous illumination, and were separated by at least 12 s of darkness to minimize photobleaching and phototoxicity. Because all optical signals were time-locked to electrical stimulation, two indicators could be imaged sequentially using a single high-speed camera without image splitting. Voltage-sensitive optical signals (ASAP2s, ASAP5-Kv, and EF-630) were acquired at a full-frame interval of 1.020 ms (∼1-kHz frame rate) using a NeuroCCD-SMQ camera (80×80  pixels; RedShirtImaging, Decatur, Georgia, United States). Calcium and glutamate signals were sampled at 2-ms (500 Hz) and 8-ms (125 Hz) intervals, respectively. Voltage imaging was performed on an Olympus BX51WI microscope equipped with two excitation light sources (475 and 630 nm), two corresponding filter sets (GFP and EF-630), and two cameras. A Dage IR-1000 camera was used for infrared differential interference contrast (IR-DIC) microscopy, whereas high-speed fluorescence imaging was performed with the NeuroCCD-SMQ camera.

### 2.5 Light Sources and Optical Filters

Calcium indicator GCaMP6f, glutamate indicator SF-iGluSnFR.A184S, and two GEVIs, ASAP2s and ASAP5-Kv, were excited using the same 475-nm light-emitting diode (LED), MIC-LED-475CG (Prizmatix Ltd., Holon, Israel) and imaged using the same optical filter set: excitation: 480/40 nm, dichroic 510 nm, and emission 535/50 nm. The light source for EF-630 was a 630-nm LED (MIC-LED-630CG, Prizmatix, Holon, Israel). Optical filter set for EF-630 included excitation (605/30 nm), dichroic (640 nm), and emission (665 LP).

### 2.6 Data Analysis

Initial data processing was performed in Neuroplex (RedShirtImaging, Decatur, Georgia, United States). Regions of interest (ROIs) were circular with a diameter of 200  μm. To estimate the number of neurons contributing to the aggregate optical signal, we assumed an effective illumination depth of 250  μm and a neuronal density of 70,000 neurons per cubic mm. Under these assumptions, each ROI corresponds to a tissue volume of 0.0079 cubic mm, containing ∼550 neurons. Optical signals were baseline-corrected, and fluorescence bleaching was removed by subtracting an exponential fit. Signal amplitudes were quantified as the difference between baseline and the peak of synaptically evoked voltage transients. Optical traces were exported using the Neuroplex function “*Save Displayed Traces as ASCII*,” which generates individual traces in simple (.txt) format. These files were imported into ClampFit (Axon Instruments, San Jose, California, United States) for analysis of ON and OFF kinetics. ON rates were obtained by fitting the “*Exponential Power*” function, whereas OFF rates were determined using the “*Exponential Sloping Baseline*” function. Time constants (tau, in ms) and ΔF/F amplitude measurements were compiled in Microsoft Excel. Statistical analysis was performed in GraphPad Prism using one-way analysis of variance (ANOVA) followed by Tukey’s *post hoc* test; and paired t test.

### 2.7 Data Availability

The datasets used and/or analyzed during the current study are available from the corresponding author upon request.

## 3 Results

**Fig. 1:**
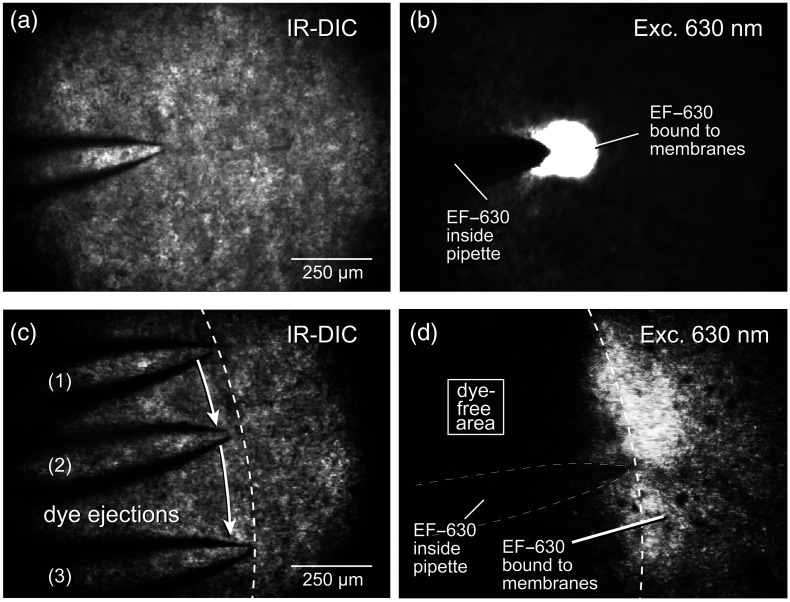
Membrane-dependent fluorescence of the VSD EF-630. (a) A glass micropipette filled with voltage-sensitive dye EF-630 (166  μM) was positioned on the surface of a mouse brain slice and visualized under IR-DIC. (b) Under 605/30 nm excitation (dubbed “630 nm”), the dye inside the pipette is nonfluorescent; however, upon pressure ejection, EF-630 binds to cell membranes and becomes fluorescent. (c) To label a defined region of tissue, the dye-filled pipette was moved along the indicated perimeter (dashed line) while applying low positive pressure. (d) The pipette trajectory becomes visible as “membrane-bound EF-630 fluorescence”. The “dye-free area” is unlikely to contribute scattered light to the signal of interest, or experience dye-induced phototoxicity.

One important but rarely emphasized feature of many VSDs is that these “fluorescent dyes” are not fluorescent in stock solution. The majority of VSDs become fluorescent only in contact with lipid membranes. To determine if the same applies to EF-630, we loaded glass micropipettes (∼2  MΩ resistance) with EF-630 dissolved in ACSF at a concentration of 166  μM. Such EF-630-loaded glass micropipettes were placed on the surface of the brain slices [Fig. 1(a)]. When excited with 630-nm light, the red emission was not observed inside the glass pipette [Fig. 1(b), “*inside pipette*”]. However, in the front end of the glass pipette, where the dye spontaneously leaked out and touched the brain slice, we observed bright fluorescence signatures [Fig. 1(b), “*EF-630 bound to membranes*”). Similarly, when a small positive pressure is used to eject EF-630 from a moving pipette [Fig. 1(c)], the pipette’s path can be traced in the fluorescence channel [Fig. 1(d)]. The jet of dye interacted with the brain slice surface and left a stripe of fluorescently labeled cells [Fig. 1(d)]. This approach based on dye ejection from a small glass pipette can potentially be useful for labeling the brain areas of special interest, while leaving other areas in the dark [Fig. 1(d), “*dye-free area*”). Such selective labeling of regions of interest (ROI) can potentially produce two benefits: (a) improvement of SNR by elimination of scattered light from neighboring areas and (b) reduction of the phototoxic effects of VSD on the biological preparation of interest (by decreasing the total brain area exposed to the dye). In summary, EF-630 is a fluorogenic, membrane-activated voltage-sensitive dye that becomes strongly fluorescent upon partitioning into the lipid membranes. Its fluorescence intensity is governed by solvatochromism, such that emission is quenched in polar environments (e.g., aqueous ACSF) and markedly enhanced in nonpolar environments, such as the lipid bilayer (Fig. 1).

### 3.1 Combined Calcium and Voltage Imaging

**Fig. 2:**
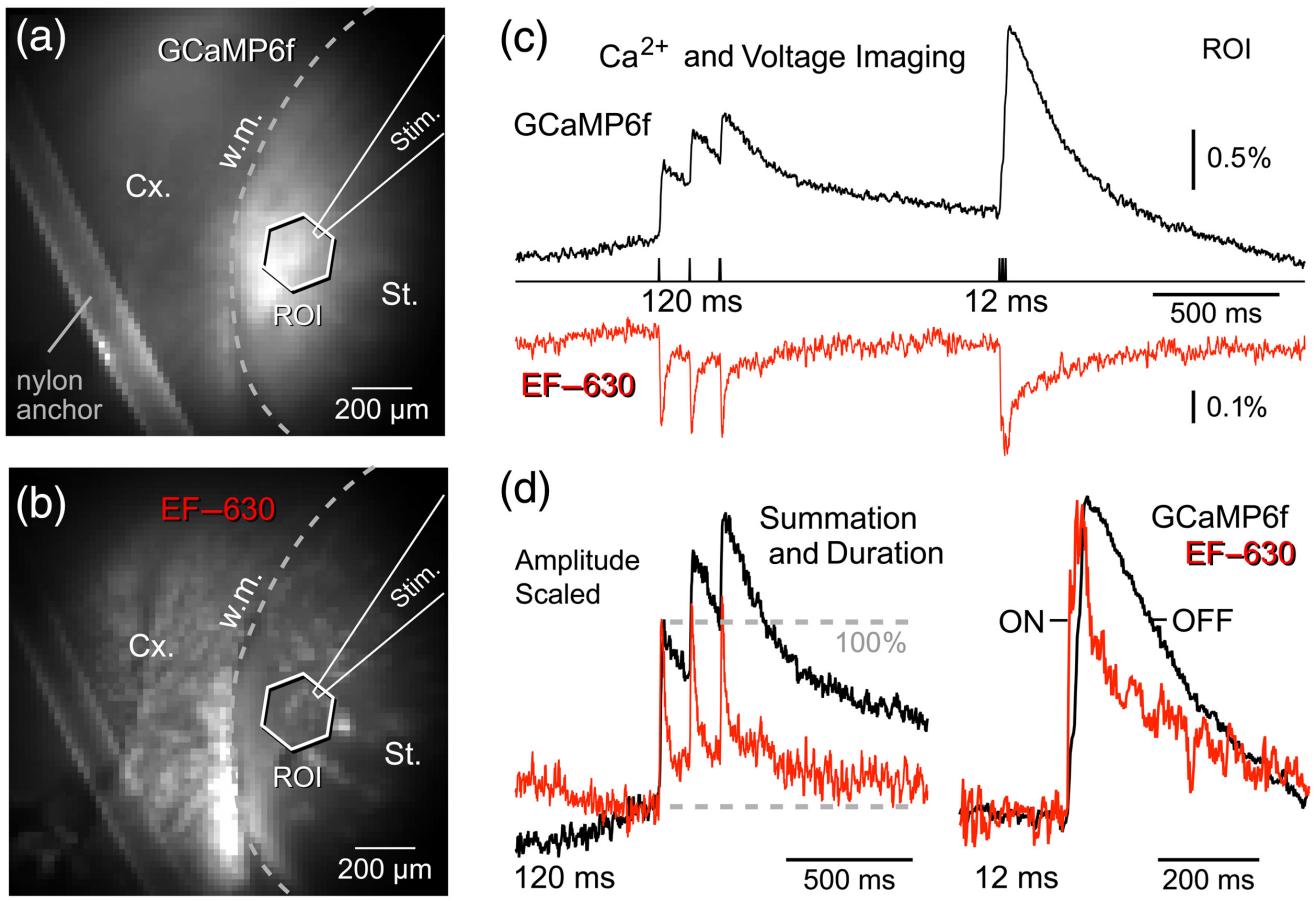
Dual-calcium voltage imaging using a GFP-based GECI (GCaMP6f) and a red-emitting VSD (EF-630). (a) Mouse striatal field of view co-labeled with GCaMP6f and EF-630. GCaMP6f expression is restricted to D1-type spiny projection neurons (D1-SPNs), whereas EF-630 uniformly labels cellular membranes without cell-type specificity. Image shown using GCaMP6f filter settings (excitation 475 nm and emission 535/50 nm). St: striatum; Cx: cortex; w.m: white matter. The dashed gray line marks the border between St. and Cx. The schematic marks the position of the extracellular stimulating electrode (Stim.). (b) The same field imaged with EF-630 filter settings (excitation 605/30 nm and emission 655/40 nm), revealing broad membrane-associated fluorescence. (c) Optical responses evoked by two stimulus triplets (interstimulus intervals: 120 and 12 ms). GCaMP6f (black, sampling rate = 500 Hz) and EF-630 (red, sampling rate = 1000 Hz) report signals with opposite polarity. (d) EF-630 traces are displayed with an inverted polarity and are amplitude-scaled, to facilitate direct comparisons between the two indicators. Essentially, the voltage-sensitive dye EF-630 exhibits faster onset (ON) and decay (OFF) kinetics, whereas GCaMP6f signals are notably slower, have longer durations (half-widths), and show marked temporal summation. The dashed gray lines mark 0% and 100% of the first optical transient. Note that the third optical transient achieves higher amplitude than the first (temporal summation).

To achieve selective expression of the calcium indicator GCaMP6f, we used a D1-Cre mouse line combined with intrastriatal injections of Cre-dependent AAVs. This strategy resulted in GECI expression restricted to D1 receptor-expressing spiny projection neurons (D1-SPNs) in the neostriatum. Following preparation of acute striatal brain slices, the voltage-sensitive dye EF-630 was applied extracellularly by bath application. Consequently, GCaMP6f fluorescence was confined to the neostriatum [Fig. 2(a)], whereas EF-630 fluorescence extended beyond the striatum into the surrounding regions, including the cerebral cortex [Fig. 2(b), Cx]. Within the neostriatum, GCaMP6f selectively labeled a single neuronal population (D1-SPNs), whereas EF-630 labeled all local cell types.

In the calcium imaging channel (green), stimulus-evoked optical signals were positive-going, reflecting increased fluorescence with rising intracellular calcium concentration [Fig. 2(c), black trace]. In contrast, in the voltage imaging channel (red), stimulus-evoked signals were negative-going, consistent with decreased fluorescence upon membrane depolarization [Fig. 2(c), red trace]. The mean amplitude of the calcium signal was 1.71±0.24% ΔF/F (n=7 ROIs, N=1 slice), whereas the mean amplitude of the voltage signal recorded from the same set of seven ROIs was 0.51±0.06% ΔF/F. Accordingly, the mean amplitude ratio (GCaMP6f/EF-630) was 3.32±0.73, indicating that calcium signals were ∼3.5 times larger than voltage signals under these conditions.

When both signals were polarity-matched (positive-going), time-aligned to stimulus onset, and plotted on the same temporal scale, marked differences in temporal dynamics became apparent [Fig. 2(d)]. Voltage transients were consistently faster, exhibiting similar rise times but narrower half-widths than calcium transients [Fig. 2(d), red versus black traces]. The mean rise time constant (tau) for voltage signals was 4.56±1.3  ms (n=7 ROIs), compared with 5.61±0.36  ms for calcium signals. Temporal differences were more pronounced during the decay phase: the mean decay-tau (tau_OFF) for voltage transients was 15.9±1.90  ms, whereas calcium transients decayed more slowly, with a mean tau of 207.29±8.27  ms (∼13-fold slower).

Together, these results demonstrate that EF-630 can be effectively combined with GFP-based GECIs for dual optical recordings. Although GCaMP6f provides larger signal amplitudes, voltage signals reported by EF-630 exhibit substantially faster kinetics in the decaying (OFF) phase, underscoring the complementary strengths of calcium and voltage imaging modalities.

### 3.2 Combined Glutamate and Voltage Imaging

**Fig. 3:**
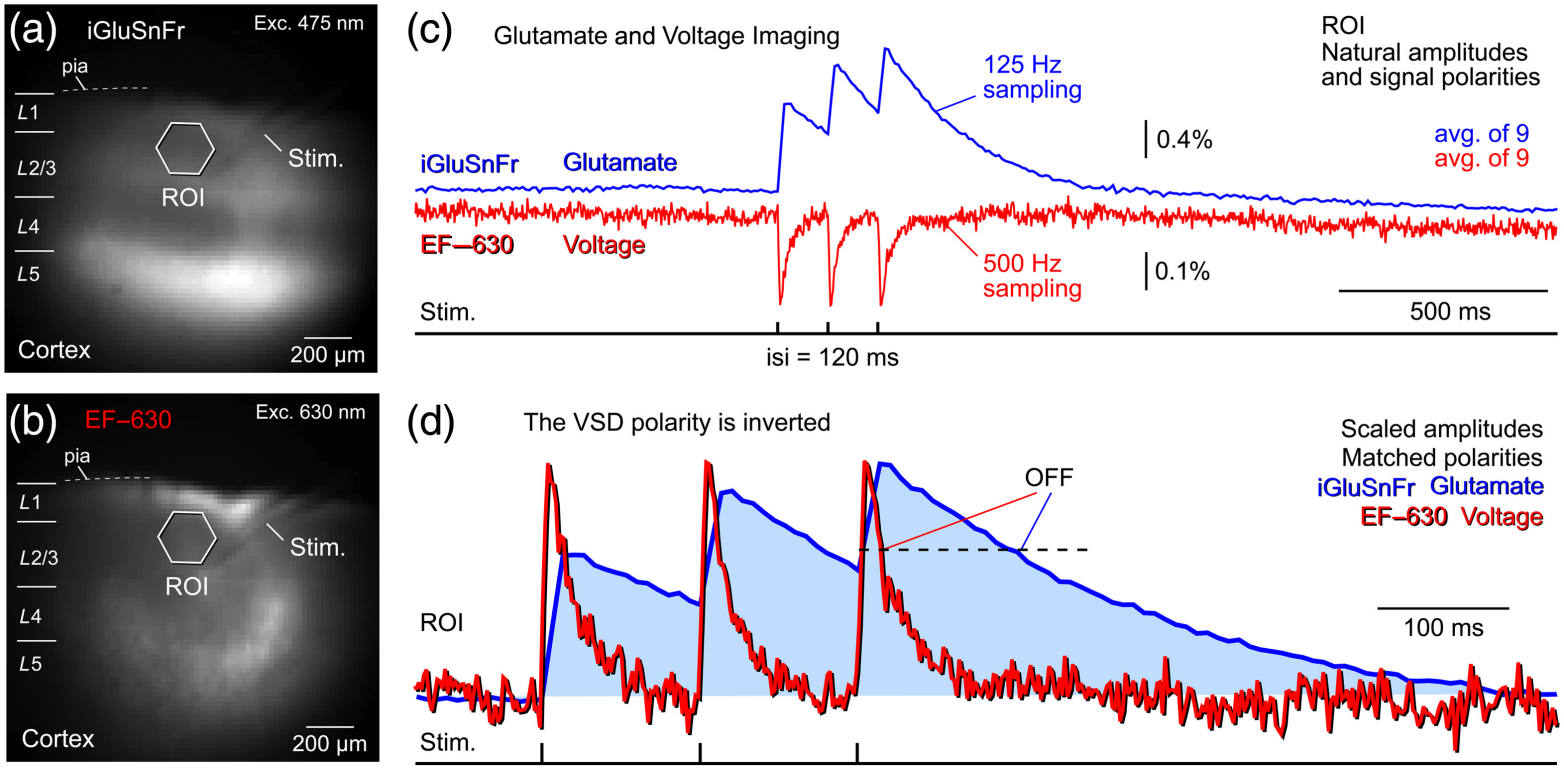
Sequential glutamate–voltage imaging with a GFP-based glutamate sensor iGluSnFr and a red VSD (EF-630). (a) Cortical field of view co-labeled with iGluSnFR and EF-630. iGluSnFR expression is restricted to cortical neurons (hSynapsin promoter), whereas EF-630 labels cellular membranes without cell-type specificity. Image acquired using GFP filter settings (excitation: 475 nm and emission: 535/50 nm). The location of the extracellular stimulating electrode is indicated (“Stim”). (b) The same field imaged using EF-630 filter settings (630 nm). (c) In the same ROI, optical responses evoked by a train of three stimulus pulses (interstimulus interval: 120 ms). iGluSnFR (blue) and EF-630 (red) report signals with opposite polarity. Each trace represents the average of nine optical sweeps. Glutamate and voltage signals were acquired at 125 and 500 Hz, respectively. (d) The voltage signal (EF-630) is shown with inverted polarity, and it is amplitude scaled for comparison with the glutamate signal (iGluSnFR). The decay (OFF) phase of the two transients is labeled on the optical trace.

To achieve expression of the glutamate indicator iGluSnFR, we used wild-type mice and injected AAVs driven by the pan-neuronal hSynapsin promoter into the neocortex. This approach resulted in the expression of the genetically encoded glutamate indicator (GEGI) in cortical neurons, including both excitatory and inhibitory populations. Following preparation of acute cortical brain slices, the voltage-sensitive dye EF-630 was applied extracellularly by bath application. Consequently, both iGluSnFR fluorescence [Fig. 3(a)] and EF-630 fluorescence [Fig. 3(b)] were detected in the cerebral cortex. In the glutamate imaging channel (green), stimulus-evoked optical signals were positive-going, reflecting increased fluorescence with rising extracellular glutamate concentration [Fig. 3(c), blue trace]. In contrast, in the voltage imaging channel (red), stimulus-evoked signals were negative-going, consistent with decreased fluorescence during membrane depolarization [Fig. 3(c), red trace]. The mean amplitude of the glutamate signal was 3.47±0.54% ΔF/F (n=23 ROIs, N=3 slices), whereas the mean amplitude of the voltage signal measured in the same ROIs was 0.34±0.02% ΔF/F. When both optical signals were polarity-matched (positive-going), time-aligned to stimulus onset, and displayed on the same temporal scale, pronounced differences in temporal dynamics were observed [Fig. 3(d)]. Voltage transients were substantially faster, exhibiting shorter rise times and narrower half-widths than glutamate transients [Fig. 3(d), red versus blue traces]. The mean rise tau for voltage signals was 4.28±0.24  ms (n=23 ROIs), compared with 11.25±0.64  ms (n=23 ROIs) for glutamate signals. Temporal differences were even more pronounced during the decay phase: the mean decay tau for voltage transients was 20.55±2.23  ms (n=23 ROIs), whereas glutamate transients decayed much more slowly, with a mean tau of 200.34±9.37  ms in the same 23 ROIs.

In summary, these results demonstrate that EF-630 can be effectively combined with GFP-based GEGIs for simultaneous voltage and glutamate imaging. Although iGluSnFR provides large-amplitude optical signals, its kinetics (both rise and decay) are considerably slower than those of voltage signals reported by EF-630, highlighting the complementary temporal and amplitude characteristics of the two imaging modalities.

### 3.3 Combining Two Voltage Indicators, GEVI and VSD

At first glance, the use of two voltage indicators within the same preparation may appear redundant, as both report membrane depolarization. However, this approach offers a unique analytical advantage: genetically encoded voltage indicators (GEVIs) can be targeted to a specific neuronal population, whereas extracellularly-applied voltage-sensitive dyes (VSDs) report aggregate membrane voltage from all cell types within the region of interest (ROI). Simultaneous acquisition of cell-type-specific and population-level voltage signals under identical stimulation conditions and from the same ROI provides complementary constraints on both conceptual and computational models of neuronal excitability and circuit function.

#### 3.3.1 ASAP2s and EF-630

**Fig. 4:**
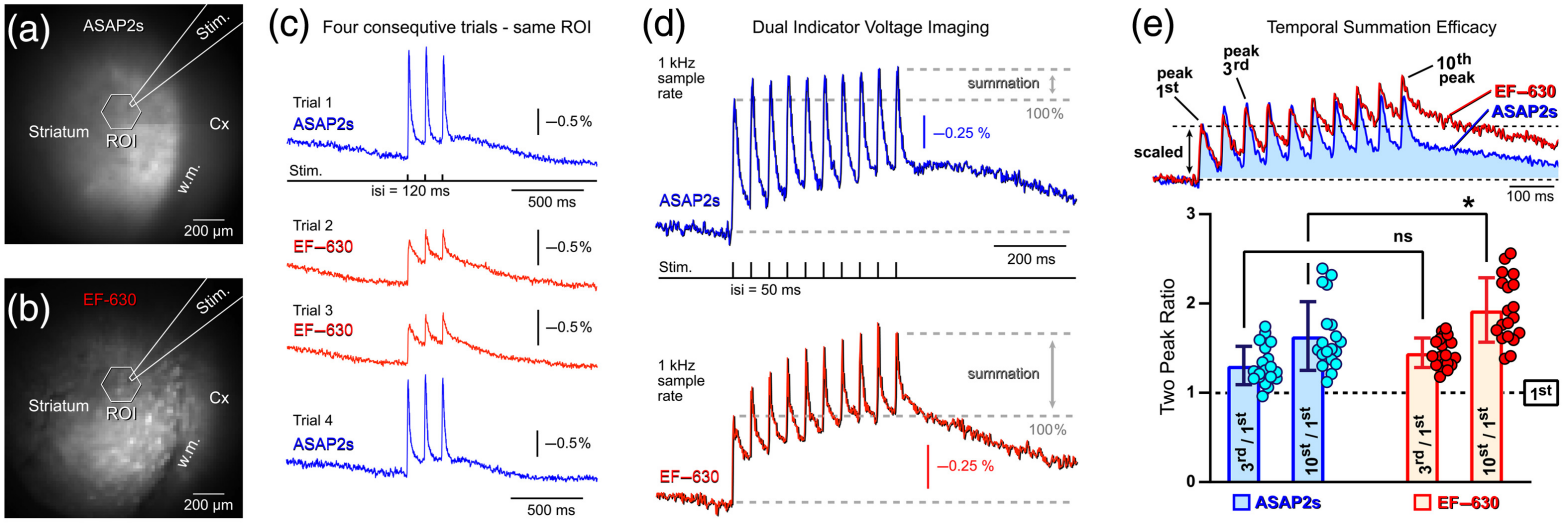
Sequential dual-indicator voltage imaging with a GFP-based GEVI (ASAP2s) and the voltage-sensitive dye (EF-630). (a) Striatal field of view (FOV) co-labeled with ASAP2s and EF-630. ASAP2s expression is restricted to D1-type spiny projection neurons (D1-SPNs), whereas EF-630 uniformly labels cellular membranes without cell-type specificity. Image acquired using GFP filter settings (Excitation: 475 nm and Emission: 535/50 nm). The position of the extracellular stimulating electrode (Stim.) is indicated schematically. (b) The same FOV imaged using EF-630 filter settings (Excitation: 605/30 nm and Emission: 655 nm long-pass). (c) Optical recordings acquired from the same ROI using alternating optical filter sets. Trials 1 and 4 were recorded using the GFP filter set (ASAP2s), whereas trials 2 and 3 were recorded using the EF-630 filter set (630 nm excitation). Apparent differences in response kinetics likely reflect differences in labeling patterns-cell-type-restricted expression of ASAP2s versus pan-membrane staining by EF-630—rather than intrinsic differences in the voltage-response kinetics of the indicators. (d) Optical signals evoked by a train of ten stimulus pulses (interstimulus interval: 50 ms). ASAP2s (blue) and EF-630 (red) report voltage-dependent optical transients of the same polarity. For visualization, signals are polarity-inverted (multiplied by −1) such that striatal depolarization appears as a positive-going transient. Temporal summation, quantified as the ratio of the 10th to the 1st optical transient (with the 1st set to 100%), is more pronounced in the EF-630 channel. In panels (c) and (d), the optical sampling rate was 1 kHz. (e) Mean ratios of the third peak relative to the first peak and the tenth peak relative to the first peak, measured within the same traces for the protein indicator (blue) and the organic dye (red). Asterisk denotes p<0.05 (one-way ANOVA); ns, not significant.

To express the voltage indicator ASAP2s selectively in D1-SPNs, we crossed ASAP2s-Flex mice with the D1-Cre line. This purely transgenic, noninvasive strategy resulted in GEVI expression at birth. Following preparation of acute striatal slices, EF-630 was applied extracellularly. ASAP2s fluorescence was confined to the neostriatum, showing a sharp border between the bright striatum and dark cortex [Fig. 4(a), Cx], whereas EF-630 fluorescence extended throughout the slice, including both striatum and cortex [Fig. 4(b), Cx]. Again, images acquired from the same field of view and focal plane exhibit distinct surface texture [Fig. 4, compare panels (a) versus (b)], reflecting differences in labeling.

In the GEVI channel, stimulus-evoked ASAP2s signals were negative-going, with fluorescence decreasing upon depolarization; signals were therefore inverted for display [Fig. 4(c), blue traces]. EF-630 signals were likewise negative-going and were similarly inverted [Fig. 4(c), red traces]. The mean ASAP2s signal amplitude was 0.65±0.05% ΔF/F (n=51 ROIs, N=4 slices), approximately twofold larger than the corresponding EF-630 signal (0.31±0.01% ΔF/F), yielding an amplitude ratio of 2.06±0.12 (n=51). Thus, ASAP2s exhibited greater optical sensitivity than EF-630.

Despite this difference in amplitude, EF-630 signals displayed more efficient temporal summation during repetitive stimulation [Fig. 4(d)]. Quantification of summation during 20 Hz stimulus trains revealed no difference between indicators for the ratio of the third to first peak; however, the ratio of the tenth to first peak was significantly larger for EF-630 [Fig. 4(e), one-way ANOVA followed by Tukey’s test]. These results indicate stronger summation in the population-level voltage signal (EF-630) than in the D1-SPN-restricted GEVI signal (ASAP2s).

Kinetic analysis showed similar rise times for EF-630 (tau_ON =5.62±0.56  ms) and ASAP2s (tau_ON =5.34±0.15  ms; n=46 ROIs). Decay kinetics, however, were slower for EF-630 (tau_OFF =26.67±2.22  ms) than for ASAP2s (tau_OFF =17.79±1.04  ms). Together, these measurements indicate that population-level voltage signals exhibit slower overall kinetics and greater summation than cell-type-restricted GEVI signals, consistent with circuit-level integration effects. In summary, EF-630 can be successfully combined with the GFP-based GEVI ASAP2s. Although ASAP2s provides larger amplitude signals and faster decay kinetics, EF-630 reports slower, more summative voltage dynamics reflecting activity across multiple neuronal populations.

#### 3.3.2 ASAP5-Kv and EF-630

To achieve cell-type-specific expression of the genetically encoded voltage indicator ASAP5-Kv,^24^ we attempted to generate transgenic mice expressing ASAP5 or ASAP5-Kv in a cre-dependent manner from the H11P locus. Only the ASAP5-Kv-injected blastocysts achieved germline transmission, so we performed analysis with these ASAP5-Kv transgenic mice only. As with ASAP2s, we crossed homozygous transgenic mice to a D1-Cre driver line to obtain selective expression of the GEVI in D1-SPNs from birth.

**Fig. 5:**
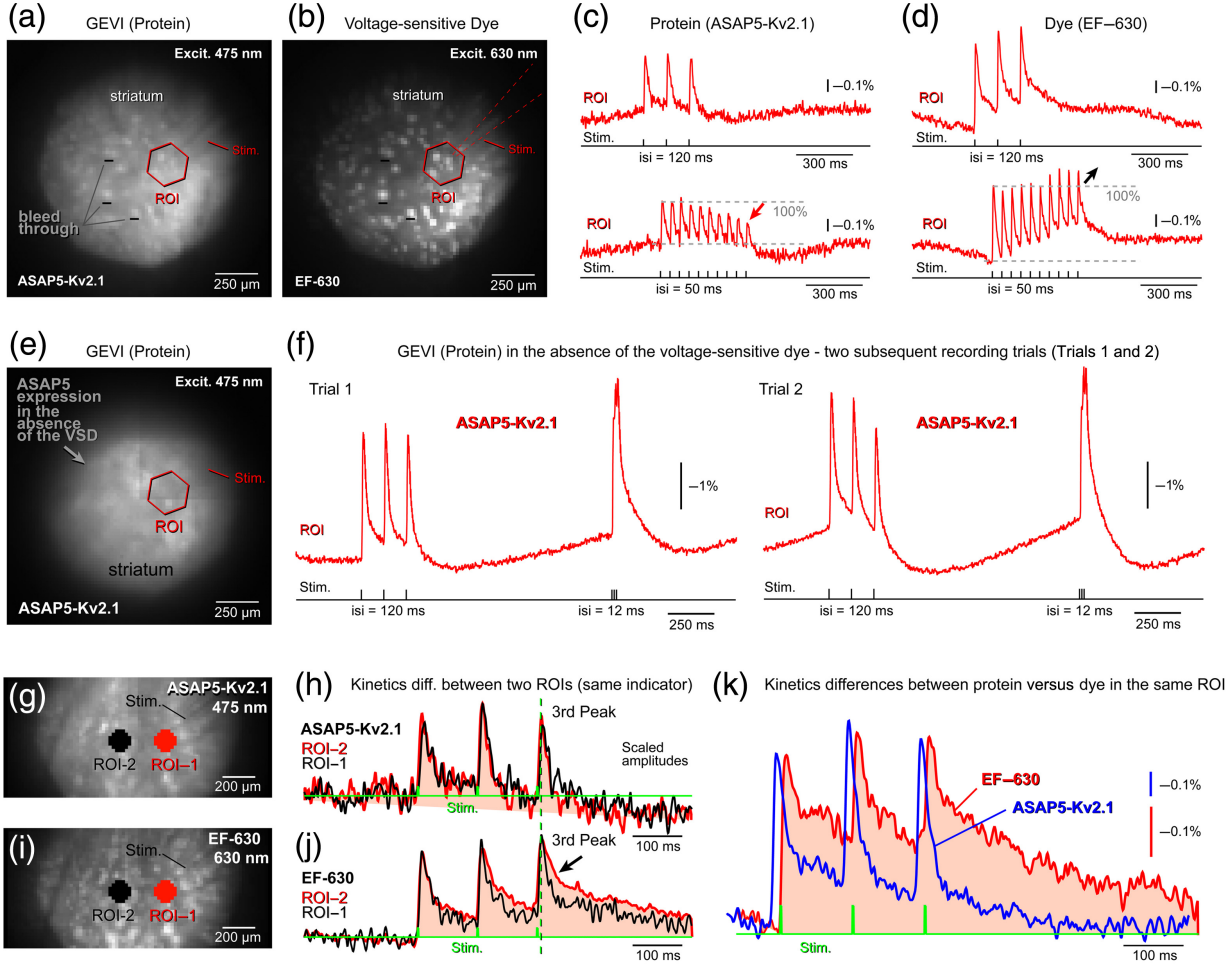
Sequential dual-indicator voltage imaging with a GFP-based GEVI (ASAP5-Kv) and the voltage-sensitive dye (EF-630). (a) Striatal field of view co-labeled with ASAP5-Kv and EF-630. ASAP5-Kv2.1 expression is restricted to the somata of D1-type spiny projection neurons (SPNs), whereas EF-630 uniformly labels cellular membranes across cell types and compartments. Image acquired using GFP filter settings (excitation: 475 nm and Emission: 535/50 nm). The positions of the extracellular stimulating electrode (Stim.) and the region of interest (ROI) are indicated schematically. (b) The same field of view imaged using EF-630 filter settings (excitation: 605/30 nm and emission: 655 nm long-pass), revealing bright puncta corresponding to axon bundles traversing the striatum. Although EF-630 emits in the red channel, dye-labeled axon bundles bleed through into the green channel; see panel (a). (c) Optical signals recorded using the GEVI channel. Top: Responses evoked by a train of three stimulus pulses (interstimulus interval [ISI]: 120 ms). Bottom: Responses evoked by ten stimulus pulses (ISI: 50 ms). The red arrow indicates synaptic depression. (d) Optical signals recorded using the EF-630 channel under the same stimulation paradigms as in panel (c). Note the differences in voltage waveform kinetics between EF-630 (this panel) and the protein-based indicator ASAP5-Kv panel (c). (e) Striatal field of view labeled with ASAP5-Kv alone (no dye present). In the absence of EF-630, the fluorescence pattern appears relatively uniform compared with the punctate pattern observed in panel A. (f) In the absence of dye labeling, stimulus-evoked ASAP5-Kv signals exhibit larger ΔF/F amplitudes. (g) Two ROIs selected within a striatal field of view co-labeled with GEVI and dye, imaged with 475 nm excitation. (h) GEVI recordings (ASAP5-Kv and 475 nm excitation) show similar voltage waveforms at both locations (ROI-1 and ROI-2). (i) The same field of view as in (g) imaged with 630 nm excitation. (j) Voltage-sensitive dye recordings (EF-630 and 630 nm excitation) reveal dissimilar voltage waveforms between ROI-1 and ROI-2, with differences most pronounced during the decay phases of the second and third peaks (arrow). (k) Within the same striatal ROI, EF-630 optical signals (red) exhibit slower decay kinetics than transients recorded with the protein-based indicator ASAP5-Kv (blue). Recordings were obtained using identical stimulation, sampling rate, and filtering parameters. For clarity, the blue trace is horizontally shifted to the left.

Acute striatal brain slices were prepared from mice aged P35-P60. Following slice preparation, the voltage-sensitive dye EF-630 was applied extracellularly by adding the dye solution directly to the recording chamber. Under this labeling strategy, ASAP5-Kv fluorescence was confined to the neostriatum [Fig. 5(a)], whereas EF-630 fluorescence extended throughout the brain slice, including the striatum, cortex, and axon bundles traversing the striatum [Fig. 5(b)]. Importantly, ASAP5-Kv selectively labeled a single neuronal subtype (D1-SPNs) and was largely restricted to the somatic compartment, whereas EF-630 labeled all cell types indiscriminately and all membrane compartments, including somata, dendrites, dendritic spines, axons, and nonneuronal membranes.

##### Signal polarity

Both ASAP5-Kv and EF-630 produced negative-going optical signals in response to depolarization. For clarity of presentation, signals from both indicators were inverted (multiplied by −1), such that depolarization is represented as a positive-going optical transient [Figs. 5(c) and 5(d)].

##### Signal amplitude

The average amplitude of stimulus-evoked ASAP5-Kv optical transients was 0.58±0.05% ΔF/F (n=21 ROIs, N=3 slices). In the same ROIs, EF-630 signals were smaller with a mean amplitude of 0.40±0.03% ΔF/F. Thus, under dual-indicator conditions, ASAP5-Kv signals were approximately 1.6-fold larger than EF-630 signals. Quantitatively, the mean amplitude ratio (ASAP5-Kv/EF-630) was 1.64±0.19 (n=21 paired measurements).

##### Impact of dye labeling on ASAP5-Kv sensitivity

When ASAP5-Kv was imaged in the absence of EF-630, optical signal amplitudes were markedly larger [Figs. 5(e) and 5(f)]. Across six dye-free brain slices, the mean ASAP5-Kv response reached 1.63±0.21% ΔF/F (n=14 ROIs, N=6 slices), representing an approximately threefold increase compared with recordings obtained in the presence of EF-630 (0.58% ΔF/F). This pronounced attenuation indicates a substantial reduction in GEVI sensitivity under dual-indicator conditions.

We attribute this effect primarily to the weak resting light intensity (RLI) of ASAP5-Kv in D1-SPN_ASAP5-Kv mice. In this line, ASAP5-Kv expression was confined to a single neuronal subtype and further restricted to the somatic compartment. Because the soma constitutes only a small fraction of the total neuronal membrane surface area, baseline fluorescence was correspondingly low. Under these conditions, the addition of EF-630 introduces substantial fluorescence bleed-through into the GEVI detection channel [Figs. 5(a) and 5(b)]. This bleed-through reduces effective ASAP5-Kv sensitivity via two mechanisms: (i) increased baseline fluorescence F, without a corresponding increase in ΔF, thereby reducing ASAP5’s fractional signal amplitude (ΔF/F); and (ii) EF-630-derived stimulus-evoked optical transients at 475-nm excitation exhibit opposite polarity to ASAP5 signals,^20^ leading to partial cancellation of voltage-dependent responses.

##### Temporal summation and kinetics

During stimulus trains delivered at interstimulus intervals of either 120 ms or 50 ms, EF-630 signals exhibited more pronounced temporal summation than ASAP5-Kv signals [Fig. 5(c) and 5(d)]. Despite this difference in summation efficacy, the rising-phase kinetics of the two indicators were broadly comparable. The average rise time constant (tau_ON) was 3.77±0.26  ms for EF-630 and 4.30±0.35  ms for ASAP5-Kv (n=21 ROIs), a statistically significant difference (paired t test, P<0.05). In contrast, the mean decay time constant (tau_OFF) did not differ significantly among indicators (EF-630: 14.69±1.76  ms; ASAP5-Kv: 16.69±1.04  ms; paired t test, P>0.05).

##### Tau_OFF variation in EF-630 recordings

In this study, EF-630 was paired with four GENIs. Despite use of the same VSD across conditions, the mean tau_OFF of EF-630 signals varied substantially between experimental groups, spanning a range from 14.69 to 26.67 ms (see above). Within individual fields of view, EF-630 recordings also exhibited subtle spatial heterogeneity. Specifically, one ROI, typically located proximal to the stimulation electrode, often displayed a slower decay phase than a more distal ROI [Fig. 5(j)]. By contrast, recordings from the same ROIs obtained with ASAP5-Kv did not show comparable spatial differences [Fig. 5(h)]. This dissociation suggests that cell types not labeled by ASAP5-Kv contribute to the spatially heterogeneous EF-630 signals. Likely contributors include D2-SPNs, striatal interneurons, invading axon terminals, and glial membranes.

##### Indicator-specific differences within the same ROI

Sequential recordings obtained from the same ROI using both voltage indicators occasionally revealed indicator-specific differences in voltage waveforms [Fig. 5(k)]. Across all such comparisons, EF-630 signals consistently exhibited slower decay kinetics than ASAP5-Kv signals. These observations reinforce the conclusion that EF-630 captures voltage dynamics from a broader and more heterogeneous cellular population, whereas ASAP5-Kv selectively reports somatic voltage changes in D1-SPNs.

### 3.4 Crosstalk Between EF-630 and GFP

**Fig. 6:**
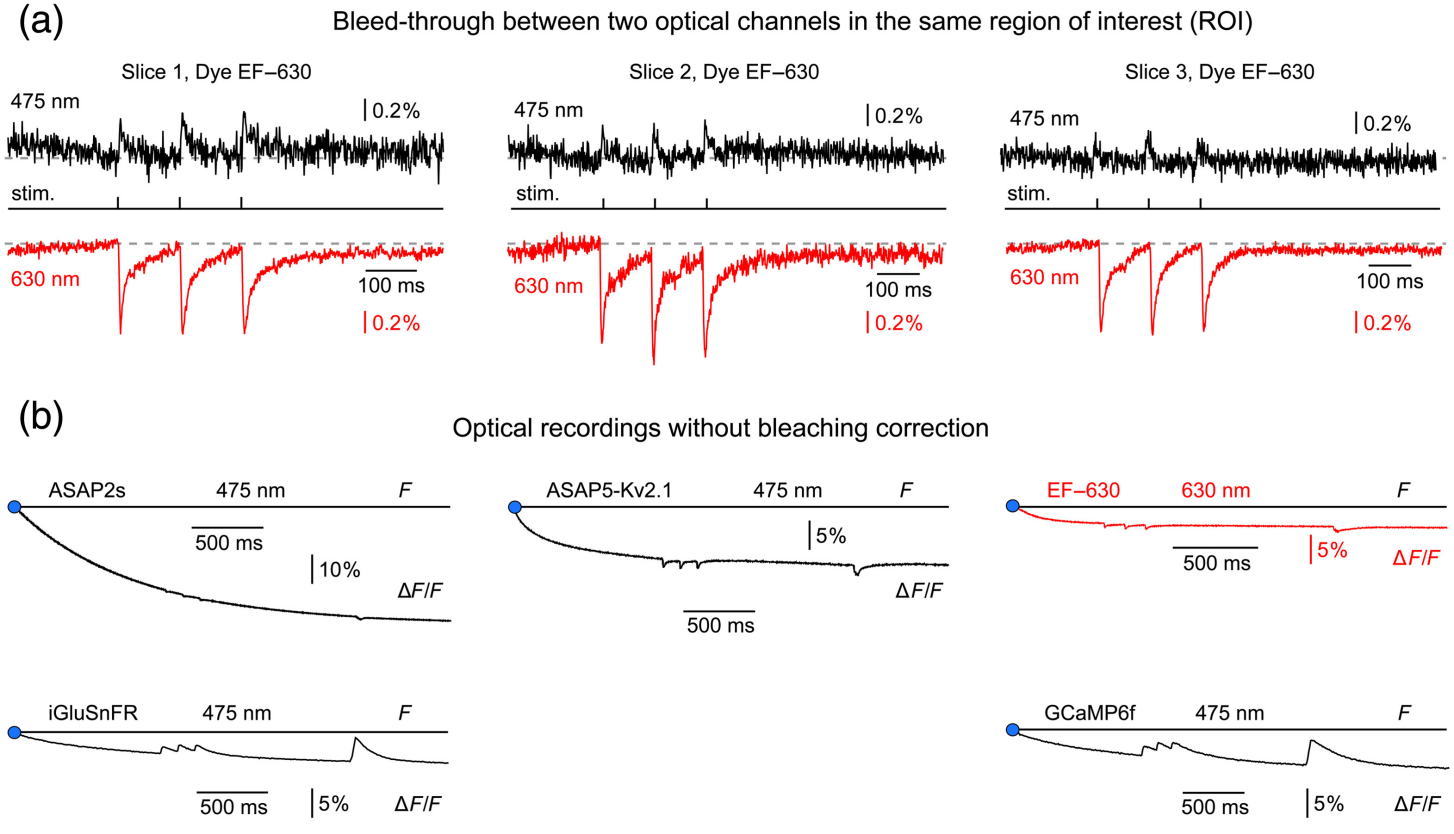
Spectral bleed-through of EF-630 into the GFP channel and indicator photobleaching. (a) Acute brain slices (Slices 1 to 3) were stained exclusively with the voltage-sensitive dye EF-630 (no additional indicators). Stimulus-evoked depolarizations were recorded optically from the same ROI using either EF-630 filter settings (red trace; excitation 605/30 nm, emission 655 nm long-pass) or GFP filter settings (black trace; excitation 475 nm, emission 535/50 nm). The GFP filter set is the same as that used in Figs. 2–5 for GFP-based activity indicators (GCaMP6f, iGluSnFR, ASAP2s, and ASAP5-Kv2.1). Identical stimulation, camera, and sampling rate were used for both recordings. No low-pass filtering was applied to preserve high-frequency noise intrinsic to optical recordings; under these identical conditions, the baseline noise amplitude (peak-to-peak) was slightly higher in the 475 nm (black) than in the 630 nm (red) channel. (b) Representative raw fluorescence traces for each indicator (no temporal filtering or bleach correction). The blue dot indicates shutter opening (LED on). The straight horizontal line denotes resting fluorescence (F) at the moment of shutter opening. All indicators, except EF-630, were illuminated with the 475-nm LED. All indicators exhibited measurable photobleaching over the 3-s acquisition window. Traces are averages of four consecutive sweeps.

The feasibility of applying this dual-voltage-indicator approach in future experiments depends critically on spectral bleed-through among the optical channels used to detect each probe. To address this point, we performed the experiments shown in Fig. 6(a). Before describing these data, we emphasize that four activity indicators (GCaMP6f, iGluSnFR, ASAP2s, and ASAP5-Kv2.1) were all recorded using identical GFP filter settings (excitation 475 nm; emission 535/50 nm). The relevant question, therefore, is the extent to which EF-630 fluorescence contaminates this GFP detection channel, which in turn depends on whether EF-630 can be excited by 475-nm illumination. To answer this question, we stained mouse brain slices with EF-630 only (no other indicators present). Evoked depolarization signals were first recorded in the standard EF-630 channel (630-nm excitation; Fig. 6(a), red trace), and then recorded again, using the GFP channel (black trace), without changing any acquisition parameters (stimulation, region of interest, or sampling). We note that the same GFP optical filter set was previously employed for GCaMP6f, iGluSnFR, ASAP2s, and ASAP5-Kv2.1 recordings in Figs. 2–5.

The black trace therefore represents the EF-630 signal detected in the GFP optical channel [Fig. 6(a), black traces; 475 nm]. No low-pass filtering was applied; the detection bandwidth was left unrestricted so that baseline optical noise and biological signals can be directly visualized.

Based on these data (n=3), we conclude that excitation of EF-630 at 475 nm produces a small positive-polarity optical signal that barely rises above the shot-noise level [Fig. 6(a), black traces]. The polarity inversion at 475 nm is expected for an electrochromic (Stark-effect) mechanism, and could, in principle, be leveraged for ratiometric recording. Fast electrochromic voltage-sensitive dyes (e.g., styryl/hemicyanine scaffolds) exhibit membrane-field-dependent shifts of their absorption spectrum, allowing excitation on either flank of the absorption band. Excitation on the blue side (e.g., 475 nm) yields signals of opposite polarity to excitation on the red side (e.g., 630 nm).

Because these crosstalk signals have positive polarity, they would introduce negative interference: that is, they would reduce the apparent amplitude of depolarization-induced optical signals measured with ASAP2s, or ASAP5-Kv2.1. In practice, this effect becomes relevant primarily when basal (resting) GEVI fluorescence is low (i.e., weak GEVI expression).

### 3.5 Indicator Bleaching

Indicator photobleaching is a key practical limitation in optical neurophysiology, as fluorescence signals decline with cumulative illumination. This decay can distort measurements during repeated stimulation or prolonged imaging, affecting quantification of neural dynamics and comparisons across trials. Characterizing bleaching behavior is therefore essential for experimental design and for assessing the usable recording window of a given probe.

In earlier figures, all optical traces were bleach-corrected (Figs. 2–5). To allow readers to examine the bleaching dynamics of the indicators used in this study, we now include optical traces without bleaching correction, for each indicator: GCaMP6f, iGluSnFR, ASAP2s, ASAP5-Kv2.1, and EF-630 [Fig. 6(b)]. No digital filtering (low-pass or high-pass) was applied [Fig. 6(b)]. Side-by-side comparison indicates that, under the present conditions (expression level and illumination intensity), ASAP2s exhibits the fastest bleaching and EF-630 the slowest [note the different voltage scales for EF-630 and ASAP2s in Fig. 6(b)]. An additional observation is that ASAP5-Kv2.1, and EF-630 display biphasic bleaching kinetics, with a rapid initial component during the first ∼500  ms after shutter opening (turning the LED on) followed by a slower exponential decay. Figure 6(b) also reveals the intrinsic optical polarity of each indicator: neuronal depolarizations produce positive-going signals in GCaMP6f and iGluSnFR, and negative-going signals in ASAP2s, ASAP5-Kv2.1, and EF-630 recordings [Fig. 6(b)].

### 3.6 Summary

Collectively, these data demonstrate that EF-630 can be combined with genetically encoded voltage indicators such as ASAP2s and ASAP5-Kv for sequential dual-indicator voltage imaging. However, the degree of functional compatibility depends strongly on the expression pattern and subcellular localization of the GEVI. In particular, the somatic restriction of ASAP5-Kv limits its ability to capture population-level voltage summation observed with the pan-membrane voltage-sensitive dye EF-630, an issue addressed further in Sec. 4.3.

## 4 Discussion

In the present study, we used population voltage imaging, an approach that reports the aggregate electrical activity of many neurons and their cellular compartments (most prominently dendrites) within the illuminated brain volume. Because voltage-sensitive signals are strongly influenced by dendritic membrane potentials,^25^^,^^26^ and because excitatory postsynaptic potentials (EPSPs) dominate dendritic voltage dynamics,^16^^,^^27^ the population voltage signal largely reflects summed synaptic activity, similar to the local field potential (LFP).^28^ The contribution of numerous neuronal elements (somata, dendrites, and axons) increases the overall signal amplitude (ΔF/F), making such aggregate signals comparatively easier to record than in single-cell voltage imaging experiments.^10^^,^^12^

### 4.1 Population Voltage Imaging

The term population imaging is traditionally applied to optical methods that lack single-cell resolution, such as wide-field or mesoscale imaging, where each pixel reports the mean activity of many neurons.^29^^–^^35^ The recorded signal therefore represents a mixed or averaged response of the underlying neuronal population. By contrast, “*multi-cell imaging*” refers to techniques that resolve the activity of individual identified neurons, producing one trace per cell.^12^^,^^36^

Although fiber photometry employs optical hardware distinct from wide-field microscopy, it lacks cellular resolution and therefore falls squarely within the category of population imaging methods.^37^^,^^38^ The imaging strategy used in the current study is therefore best understood as a population-level approach without single-cell information. Loss of single-cell resolution can result from several methodological factors, including: (1) dense labeling of neurons with fluorescent indicators,^14^ (2) nonselective expression throughout somata, dendrites, and axons, (3) lack of axial sectioning (we used single-photon wide-field illumination), (4) low magnification, producing a comparatively large focal volume (we used a 10x objective), and (5) light scattering within brain tissue.

In our experiments, synaptic stimulation activated many neurons simultaneously, and activity from numerous cells was projected onto individual camera pixels. Consequently, the optical signals resemble LFPs, which are themselves dominated by synaptic potentials.^28^ Even under a conservative assumption of substantial light scattering (e.g., each pixel contributing ∼50% of its signal to its nearest neighbor) the spatial resolution of our voltage imaging is estimated at 50 to 100  μm,^29^ a value modestly better than the 200 to 400  μm spatial spread characteristic of LFPs.^28^

### 4.2 Optical Signal Polarity

Unlike LFPs, which measure extracellular voltage and therefore exhibit polarity reversals as depolarization waves propagate relative to the recording electrode,^39^ optical voltage indicators report transmembrane voltage directly. Accordingly, the sign of the optical signal is fixed and intuitive: depolarization and hyperpolarization produce opposite signal changes that directly reflect the underlying membrane potential, independent of propagation direction. This propagation-invariant sign makes population voltage imaging (both VSD- and GEVI-based) a powerful complement to electrophysiological population measures such as the LFP. Note: For some voltage indicators (e.g., EF-630, ASAP2s, and ASAP5-Kv), the raw optical signal is inverted for display purposes. In these cases, multiplying traces by −1 ensures a more natural display, in which depolarization appears as a positive-going signal and hyperpolarization as a negative-going signal.

### 4.3 Weakening of the GEVI Optical Signal When Dye is Present

In the presence of EF-630 labeling, ASAP5-Kv produced significantly smaller optical signals [Fig. 5(c)] than the same GEVI recorded in the absence of EF-630 [Fig. 5(f)]. An initial indication of the underlying mechanism is provided by comparing images acquired from the same field of view using different optical filter sets [Fig. 5(a) versus Fig. 5(b)]. Because the resting light intensity (RLI) of ASAP5-Kv in this experiment is intrinsically low, imaging required both increased excitation intensity and elevated camera gain. Under these high-gain, high-illumination conditions, fluorescence from EF-630-labeled axon bundles began to bleed into the green GEVI detection channel [Fig. 5(a)], contaminating the ASAP5-Kv signal.

The low RLI of ASAP5-Kv is attributable to its subcellular localization. ASAP5-Kv is restricted to the somatic membrane, which represents only ∼15% of the total neuronal membrane surface area. In contrast, dendrites and axons account for ∼85% of neuronal membrane, and the absence of indicator expression in these compartments substantially reduces total tissue fluorescence. This effect is further compounded by the fact that ASAP5-Kv expression was confined to a single neuronal population (D1-SPNs), further limiting circuit-level RLI. Moreover, at 475-nm excitation (the optimal wavelength for ASAP5-Kv), the VSD EF-630 generates voltage-dependent signals of opposite polarity to ASAP5-Kv,^20^ leading to signal cancellation between the two indicators and a consequent reduction in apparent signal amplitude [Fig. 5(c)]. In contrast, when GEVIs are robustly expressed across all neuronal compartments, particularly in dendrites, as exemplified by ASAP2s (Fig. 4), the resulting high RLI permits imaging with lower excitation intensity and reduced camera gain. Under these conditions, EF-630 fluorescence does not bleed into the GEVI channel [Fig. 4(a)] and therefore does not measurably degrade GEVI signal quality [Fig. 4(c), blue trace].

### 4.4 Discrepancy in Temporal Summation Between Protein and Dye

Biophysically, in neurons, temporal summation arises from several membrane- and circuit-level factors, including: (a) the neuronal membrane time constant, (b) incomplete repolarization among stimuli, (c) overlapping synaptic conductances, and (d) incomplete recovery of voltage-gated channels.

In population voltage imaging, stimulus trains typically produce a small ΔF/F response to the first pulse, followed by progressively larger responses to subsequent pulses [Fig. 4(d), red trace]. Temporal summation in optical voltage recordings reflects the progressive accumulation of membrane depolarization when individual voltage transients fail to fully decay before the next stimulus. At sufficiently high stimulation frequencies, each evoked depolarization overlaps with the next, yielding a stepwise increase in optical signal amplitude across the train. Importantly, this phenomenon corresponds to genuine membrane voltage integration rather than an artifact of the optical system, but see Ref. 40. In dual whole-cell and population voltage imaging recordings, in the patched neuron, synaptically-evoked depolarizations do not summate, whereas the population voltage imaging signal exhibits evident summation (their figures 2 and 3 in Ref. 40).

In our current recordings, we observed clear differences in temporal summation efficacy between ASAP2s and EF-630 [Fig. 4(e)], as well as between ASAP5-Kv and EF-630 [Figs. 5(c) and 5(d)]. Such differences are expected in optical electrophysiology and usually reflect a combination of indicator kinetics, neuronal biophysics, and tissue-level sampling rather than simply differences in the underlying neuronal summation. Below, we outline three well-established mechanisms that predict stronger apparent temporal summation in GEVIs than in VSDs, even when both indicators sample the same neuronal population under identical stimulation conditions.

1. Slower kinetics of GEVIs (integration by the sensor itself: Many GEVIs exhibit slower on- and off-kinetics than VSDs, including longer fluorescence decay time constants. VSDs, by contrast, typically respond on sub-millisecond timescales and therefore track voltage changes with minimal artificial accumulation.^41^ GEVI signals can thus integrate across stimulus intervals simply because the indicator persists in a bright state longer than the underlying voltage transient.^13^^,^^42^
2. Preferential GEVI sensitivity to subthreshold depolarization: Temporal summation reflects the integration of subthreshold EPSPs and dendritic depolarization. GEVIs often show enhanced sensitivity to slow, small-amplitude depolarizations, particularly in dendrites,^27^^,^^43^ whereas VSD signals tend to be dominated by fast population spikes.^6^^,^^44^ Consequently, GEVIs may emphasize integrative dendritic processes, whereas VSDs may highlight fast, spike-driven dynamics.
3. Differential interaction with extracellular field stimulation: Field stimulation activates axons, synapses, and polarizes membranes to varying degrees.^45^ VSDs often report axonal volleys and fast population spikes with high sensitivity,^6^^,^^46^ whereas GEVIs preferentially capture postsynaptic depolarization and dendritic integration.^27^^,^^43^ Because temporal summation is inherently a postsynaptic process, GEVIs would be expected to show stronger summation than VSDs.
  Despite these theoretical expectations, our data revealed the opposite pattern: ASAP2s exhibited less temporal summation than EF-630 under identical stimulation in the same region of interest [Figs. 4(d) and 4(e)]. Two additional factors likely account for this finding:
4. Nonlinear fluorescence responses in GEVIs: Several GEVIs display nonlinear ΔF/F responses at depolarized potentials, including state-dependent saturation and slow recovery from bright states.^47^ Such nonlinearities can produce apparent plateauing or attenuation of summation during stimulus trains [Figs. 4(d) and 5(c)]. In our recordings, ASAP2s signals saturated around the third to fourth response and showed minimal additional growth thereafter [Fig. 4(d), blue trace). In contrast, VSDs behave much more linearly with respect to membrane voltage,^48^ preserving the additive structure of individual depolarizations [Fig. 4(d), red trace).
5. Differences in membrane targeting and sampling across cell types: GEVIs are genetically targeted to specific neuronal populations and are typically enriched in somatic and dendritic membranes.^49^^,^^50^ VSDs, however, indiscriminately label all cellular membranes, including glial cells, axons, and vasculature.^30^^,^^46^ This broad staining increases the contribution of passive membrane compartments that decay slowly and desynchronize relative to fast neuronal signals. As a result, VSD recordings may show broader, slower-decaying population responses, which can produce stronger apparent summation [Fig. 4(e), red trace, 10th peak]. For example, Kojima et al. and Pal et al. revealed a surprisingly strong glial contribution to the voltage population signal.^39^^,^^51^ In essence, the heterogeneous membrane pool sampled by VSDs leads to prolonged fluorescence transients that overlap across stimuli.

Together, these considerations highlight that differences in temporal summation between GEVIs and VSDs arise from a complex interplay of sensor kinetics, fluorescence nonlinearities, membrane targeting, and population sampling, rather than solely from differences in neuronal integration. A rigorous interpretation of summation dynamics thus requires careful attention to the biophysical and optical properties of each indicator.

### 4.5 GEVI Response Time

The two GEVIs employed in the current study (ASAP2s and ASAP5) exhibit relatively slow rise dynamics (tau ON) in population-level optical recordings (Figs. 4 and 5). Importantly, these measurements reflect the spatiotemporal summation of synaptically evoked depolarizations across hundreds of dendritic branches expressing the indicator. Each branch receives excitatory postsynaptic potentials (EPSPs) with variable onset times and propagation delays, resulting in an optical signal that represents a temporally dispersed population average rather than a single membrane event. Consequently, the GEVI response times reported here (Figs. 4 and 5) should not be interpreted as intrinsic sensor kinetics at the single-cell level. Instead, they are shaped by network-level dynamics, dendritic filtering, and synaptic integration, all of which broaden the apparent rise time.

Under conditions where membrane voltage is tightly controlled, such as voltage-clamp step protocols in individual cells, the intrinsic kinetics of these indicators are substantially faster. Reported fast-tau ON values are as follows: <5  ms for ASAP2s,^41^^,^^52^ and <1  ms for ASAP5.^24^ These comparisons highlight that the slower dynamics observed in the present study primarily arise from biophysical and circuit-level factors, rather than limitations of the GEVI sensors themselves.

### 4.6 Potential Use of Dual Protein + Dye Voltage Imaging

In neurobiological experiments, combining a genetically encoded voltage indicator (GEVI) with a voltage-sensitive dye (VSD) can provide a more comprehensive understanding of the circuit under investigation.^1^ Because a GEVI typically labels only one cell type, and sometimes only a specific cellular compartment (e.g., soma; ASAP5-Kv), its readout is inherently selective. In contrast, an extracellularly-applied (e.g., bath-applied) VSD indiscriminately labels all cell types and all major neuronal compartments, including soma, proximal and distal dendrites, dendritic spines, the axon initial segment, axon trunk, and axon terminals. Computational models of active neural networks, particularly those that incorporate detailed multicompartmental neuron representations, have become essential for elucidating circuit organization and the underlying logic of information processing.^53^^,^^54^ Having access to two complementary voltage waveforms, one derived from a specific genetically targeted population (e.g., D1-SPN, Fig. 4(c), blue trace], and the other representing the aggregate activity of all cells [Fig. 4(c), red trace], can provide valuable constraints for such models and potentially improve the interpretability of circuit-level dynamics.

## 5 Conclusion

We demonstrate that the far-red voltage-sensitive dye EF-630 (emission 665 to 700 nm) can be successfully combined with several GFP-based genetically encoded indicators of neuronal function, including the calcium indicator GCaMP6f, the glutamate indicator iGluSnFR, and the voltage indicators ASAP2s, and ASAP5-Kv (emission ∼500  nm). Because GCaMP6f primarily reports neuronal output in the form of action potentials (evoked somatic calcium influx), whereas EF-630 predominantly reports subthreshold depolarizations within dendritic neuropil, simultaneous GCaMP6f and EF-630 imaging within the same region of interest (ROI) can reveal complementary aspects of neuronal dynamics that neither indicator provides alone. Similarly, iGluSnFR reports neuronal input by detecting extracellular glutamate released from presynaptic afferents, whereas EF-630 reflects postsynaptic membrane depolarization in dendrites. Combining iGluSnFR and EF-630 imaging therefore enables simultaneous visualization of synaptic input (glutamatergic afferents) and the resulting subthreshold postsynaptic responses (EPSPs) within the same ROI, providing a richer view of synaptic processing. Finally, dual-voltage imaging using both a cell type-specific GEVI, and the broadly labeling VSD EF-630, can be particularly informative for computational neuroscience: the GEVI isolates activity from a targeted population (e.g., D1-SPNs, Fig. 4), whereas EF-630 reports the aggregate voltage dynamics of all neurons within the field of view. This pairing constrains the interpretation of cell type-specific signals within the broader network context. Spectral bleed-through from EF-630 into GFP-based indicator recordings is negligible under typical conditions but can introduce a small negative bias in depolarization-evoked GEVI signals when basal GEVI fluorescence is weak. Bleaching dynamics vary across indicators; these differences define the practical recording window and should be considered when selecting probe combinations and illumination regimes for dual-indicator experiments. In dual “protein + dye” experiments, however, it is important to recognize that the addition of a VSD may reduce the optical signal from the protein indicator even when spectral overlap is minimal. This effect is especially pronounced when the protein’s resting fluorescence is inherently weak, for example due to low brightness, low expression level, or localization to a small membrane area (e.g., soma-restricted expression).

## Data Availability

The datasets used and/or analyzed during the current study are available from the corresponding author upon request.
